# Effect of Majapahit (*Crescentia cujete* L.) fruit powder on the immune profile of *Litopenaeus vannamei* after infection with *Vibrio* spp

**DOI:** 10.14202/vetworld.2021.1480-1486

**Published:** 2021-06-10

**Authors:** Sri Rahmaningsih, Riska Andriani, Hernik Pujiastutik

**Affiliations:** 1 Department of Fisheries Science, Faculty of Fisheries and Marine, University of PGRI Ronggolawe, Tuban 62381, East Java, Indonesia; 2 Department of Biology, Faculty of Mathematics and Natural Sciences, University of PGRI Ronggolawe, Tuban 62381, East Java, Indonesia; 3 Department of Biology Education, Faculty of Teacher Training and Education, University of PGRI Ronggolawe, Tuban 62381, East Java, Indonesia

**Keywords:** *Crescentia cujete* L, hemocytes, immune response, phagocytosis

## Abstract

**Background and Aim:**

The use of bioactive compounds is a promising tool to improve shrimp health regarding vibriosis. This study aimed to determine the effect of the dietary Majapahit (*Crescentia cujete* L.) fruit powder on the cellular immune response of vannamei shrimp (*Litopenaeus vannamei*) infected with *Vibrio harveyi*, *Vibrio alginolyticus*, and *Vibrio parahaemolyticus*.

**Materials and Methods:**

Twelve vannamei shrimp (aged 1 month) were randomly divided into four groups (n=3). Vannamei shrimp received experimental feed with different concentrations of Majapahit fruit powder for 20 days: Group A, 0%; Group B, 3.04%; Group C, 6.08%, and Group D, 9.12%. Subsequently, vannamei shrimp was infected with *V. harveyi*, *V. alginolyticus*, and *V*. *parahaemolyticus*. The total hemocytes, total differential hemocytes (hyaline, semi-granular, and granular cells), and phagocytic activity were assessed. Data were analyzed through analysis of variance (p<0.05) using SPSS ver. 24 for Windows.

**Results:**

Majapahit fruit powder at a dose of 3.04% increased the number of hyaline cells of *L. vannamei* after infection with *Vibrio* spp. Supplementation of the feed formula with Majapahit fruit powder at a dose of 3.04% increased the number of semi-granular and granular cells compared with the control. Furthermore, Majapahit fruit powder at doses of 3.04% and 6.08% increased the hemocytes compared with a dose of 9.12%. The phagocytic activity of *L. vannamei* after infection with *Vibrio* spp. tends to increase after supplementation with 3.04% Majapahit fruit powder.

**Conclusion:**

This study showed that the addition of *C. cujete* L. powder to the feed formula increased the cellular immune response. The most effective dose ranged from 3.04% to 6.08%.

## Introduction

Whiteleg shrimp or vannamei shrimp (*Litopenaeus vannamei*) is among the cultivated fishery commodities with the highest production rate among other types of shrimp. To meet international market demands, it is necessary to increase and develop the effectiveness and efficiency of business cultivation [1]. According to the Ministry of Marine Affairs and Fisheries, *L. vannamei* production has increased yearly from 2009 to 2013, reaching 390,278.00 tons in 2013 [2]. Vibriosis is a problem in the cultivation of *L. vannamei* that is caused by *Vibrio* spp. *Vibrio* spp. are pathogenic organisms that spread quickly and cause death at a rate up to 85% [3]. In general, several diseases that attack shrimp can be treated with antibiotics. However, the continuous administration of antibiotics leads to the spread of pathogens because of antibiotic resistance [4]. This condition requires an alternative treatment, such as treatment with natural ingredients.

The pulp of Majapahit (*Crescentia cujete* L.) fruit is one of the medicinal ingredients that can inhibit the development of bacteria because of the presence of ­bioactive compounds, such as carotenoids, phenolics, alkaloids, pectins, tannins, flavonoids, and terpenoids. It also contains coumarins such as aegeline, aegelenine, marmelin, *O*-methyl halfordinol, alloimperatorin, furocoumarins, psoralen, *O*-isopentenyl halfordinol, and marmelosin [5]. However, the flesh fruit of Majapahit contains phenolic compounds that act as healing agents for wounds caused by bacterial infection by damaging and penetrating the bacterial cell walls. Moreover, it contains flavonoids, which could prevent oxidation and inhibit the spread of wounds properly. Several *in silico* studies have shown that the use of Majapahit plant extracts, including the stem, leaves, and fruit, could inhibit the growth of *Vibrio harveyi* [6]. Furthermore, the use of Majapahit plant extracts boosts the immune system in Sangkuriang catfish infected with *Aeromonas hydrophila* [7,8].

The prevention of vibriosis caused by bacterial attack, especially by *Vibrio* spp., is urgently needed. The use of natural ingredients at a proper dosage will help maintain shrimp health. Therefore, this study aimed to examine the effect of supplementation of the feed formula with Majapahit fruit powder on the immune response and prevention of vibriosis disease in *L. vannamei*.

## Materials and Methods

### Ethical approval

This study does not need ethical approval.

### Study period and location

This research was conducted from March until October 2019 at the Laboratory of Fisheries and Marine Faculty, PGRI Ronggolawe University, Tuban.

### Preparation of experimental feed

The experimental feed of *L. vannamei* consisted of 39% protein, 4.2 kcal/g energy, and Majapahit fruit powder. Feed ingredient composition is shown in Table-1. The formula of the experimental feed of *L. vannamei* is shown in Table-2. According to Ekawati *et al*. [9], shrimp require a protein content of 30-40% to grow appropriately. A proximate analysis of vannamei shrimp feed was performed and the feed was administered according to the size of the shrimp.

**Table-1:** Feed ingredients composition.

Ingredients	Rebon flour (%)	Tapioca flour (%)	Majapahit fruit powder (%)
Protein	6.66	1.39	3.27
Fat	4.11	0.45	0.79
Carbohydrate	4.03	88.58	77.76
Ash	11.79	1.44	11.05
Crude fiber	3.04	0.32	12.80
Water	19.40	8.13	16.55

**Table-2:** *Litopenaeus vannamei* feed formula trial.

Material (Gram)	Treatments			

	A (0%)	B (3.04%)	C (6.08%)	D (9.12%)
Rebon flour	64.20	64.20	64.20	64.20
Tapioca flour	12.58	10.09	8.11	6.73
Majapahit fruit powder	0.00	3.04	6.08	9.12
Fish oil	3.75	3.75	3.75	3.75
Corn oil	6.50	6.50	6.50	6.50
Vitamin mix	2.70	2.70	2.70	2.70
Mineral mix	2.00	2.00	2.00	2.00
CMC	8.22	7.75	6.67	5.00
Total	100	100	100	100

### Experimental animals, diet, and bacterial infection

*L. vannamei* that were used in this study (1 month of age and weighing 0.02 g) were obtained from pond farmers in the Tuban Regency. Vannamei shrimp were kept in an aquarium with a size of 60×30×30 cm filled with water at 30 ppt salinity and a stocking density of 10 fish/40 L of water. Twelve vannamei shrimp were randomly divided into four groups (n=3). Vannamei shrimp received experimental feed with different concentrations of Majapahit fruit powder for 20 days, as follows: Group A, 0%; Group B, 3.04%; Group C, 6.08%; and Group D, 9.12%. The dose of Majapahit fruit powder was selected based on previous research by Ekawati *et al*. [9]. The experimental feed was given for 5% of body weight at 08:00 am and 04:00 pm GMT+7.

After receiving the experimental feed for 20 days, *L. vannamei* were infected with *Vibrio harveyi*, *Vibrio alginolyticus*, and *Vibrio parahaemolyticus*. The total density of bacteria was 10^7^ cells/mL, which were then used for infection by immersion for 24 h. *L. vannamei* were kept in an aquarium with a size of 60×30×30 cm filled with water at a salinity of 30 ppt and a stocking density of three individuals/10 L of water. At the end of the experiment, before and after infection, shrimp hemolymph was collected using a 1 mL syringe that had been filled with 10% Na-citrate (as an anticoagulant), pH 7.2 with a hemolymph ratio of 1:1.

### Cellular immune response test

#### Differential hemocyte count (DHC)

The differential count of hemocyte cells, including hyaline, semi-granular, and granular cells, was performed based on morphological criteria using light microscopy observation at 1000×.

#### Total hemocyte count (THC)

Fish blood was placed into a tube up to 0.5 μl and trypan blue was added at a ratio of 1:1. The THC was then calculated using a hemocytometer through light microscopy at 400×.

The calculation formula was as follows:


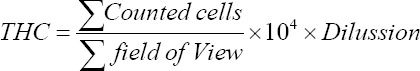


### Phagocytic activity

Blood (0.1 mL) was collected and placed into Eppendorf tubes, followed by mixing of 0.1 mL of PBS with 0.1 mL of *Vibrio* spp. (10^7^ cells/mL). Subsequently, 0.1 mL was transferred to a 2 mL tube containing blood and homogenized. The sample was incubated for 45 min at room temperature (20-25^o^C). A drop of the mixture was then smeared on a glass slide and air-dried. The slide was rinsed using 95% alcohol, then with water for 3 min. Finally, the slide was stained with Giemsa for 20 min and rinsed with distilled water.

The phagocytosis activity was observed under a microscope and measured by the following formula:


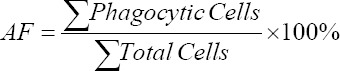


### Statistical analysis

The data were analyzed with tests for normality and homogeneity. Analysis of variance was used with a threshold of 5%. A least significant difference test using Duncan’s multiple range test was then used to assess differences between treatments using IBM SPSS Statistics ver. 24 for Windows (IBM Corp., New York, USA).

## Results

### DHC

#### Hyaline cells

Numbers of hyaline cells of *L. vannamei* in treatment B (dose 3.04%) after 20 days were elevated (79.56%). The lowest numbers, 76.21% were, found with treatment C (dose 6.08%). Supplementation with Majapahit fruit powder showed a significant effect (p<0.05) on hyaline cells counts compared to controls without Majapahit fruit powder.

**Figure-1:**
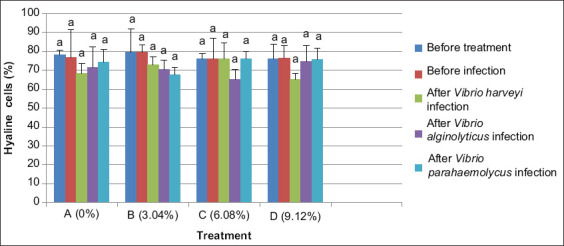
The number of hyaline cells of vannamei shrimp (*Litopenaeus vannamei*) before and after infected with *Vibrio harveyi, Vibrio alginolyticus*, and *Vibrio parahaemolyticus* and treatment with Majapahit (*Crescentia cujete* L.) fruit powder. Data were indicated as mean value±SD. Graphs with different notations showed significantly different (p<0.05).

Hyaline cell numbers of *L. vannamei* in all ­treatments decreased after infection with *V. harveyi, V. alginolyticus*, and *V. parahaemolyticus*. The lowest hyaline cell counts were observed in Group C (65.21%). Cells counts after feeding and infection with *V. alginolyticus* and *V. parahaemolyticus* in each treatment were not different. However, Majapahit fruit powder at a dose of 3.04% (B group) increased number of hyaline cells (Figure-1).

#### Semi-granular cells

The highest semi-granular cell counts in shrimp maintained for 20 days with experimental feed were observed in treatment C (dose 6.08%) with values as high as 19.69%. The lowest numbers, about 13.36%, were found for treatment B (dose 3.04%). Addition of Majapahit fruit powder to feed produced a significant increase (p<0.05) in numbers of semi-granular cells.

**Figure-2:**
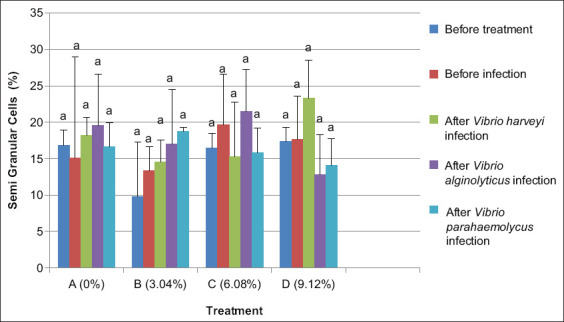
The number of semi-granular cells of vannamei shrimp (*Litopenaeus vannamei*) before and after infected with *Vibrio harveyi, Vibrio alginolyticus*, and *Vibrio parahaemolyticus* and treatment with Majapahit (*Crescentia cujete* L.) fruit powder. Data were indicated as mean value±SD. Graphs with different notations showed significantly different (p<0.05).

After infection with *V. harveyi* and *V. parahaemolyticus* for 24 h, numbers of semi-granular cells decreased reduction in treatment C (dose 6.08%). After *V. alginolyticus* infection, semi-granular cell counts decreased in treatment D (dose of 9.12%). However, treatment A (dose of 0%) or without the addition of Majapahit fruit powder showed increasing numbers of semi-granular cells. Overall, treatments did not differ significantly after feeding and infection with *V. harveyi, V. alginolyticus*, and *V. parahaemolyticus* (Figure-2).

### Granular cells

**Figure-3:**
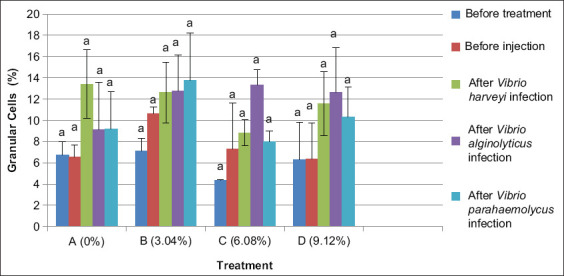
The number of granular cells of vannamei shrimp (*Litopenaeus vannamei*) before and after infected with *Vibrio harveyi, Vibrio alginolyticus*, and *Vibrio parahaemolyticus* and treatment with Majapahit (*Crescentia cujete* L.) fruit powder. Data were indicated as mean value±SD. Graphs with different notations showed significantly different (p<0.05).

*L. vannamei* showed the highest numbers of granular cells (10.61%) for treatment B (dose of 3.04%) after 20 days with experimental feed. The lowest numbers were observed for treatment A (dose 0%), up to 6.54%. The addition of Majapahit fruit powder significantly increased granular cell numbers compared to the group without the powder (p<0.05). After infection with *Vibrio* spp. for 24 h, granular cell counts showed the highest counts in treatment B (dose 3.04%). Overall, treatments did not differ significantly after feeding and infection with any of the *Vibrio* strains (Figure-3).

### THC

**Figure-4:**
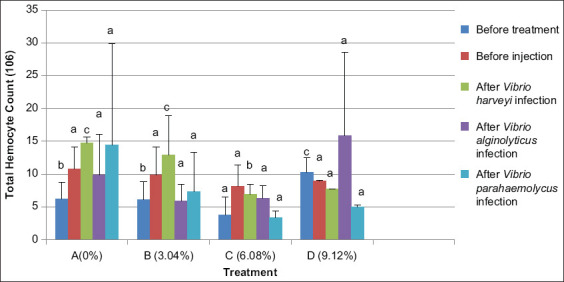
The total hemocytes of vannamei shrimp (*Litopenaeus vannamei*) before and after infected with *Vibrio harveyi, Vibrio alginolyticus*, and *Vibrio parahaemolyticus* and treatment with Majapahit (*Crescentia cujete* L.) fruit powder. Data were indicated as mean value±SD. Graphs with different notations showed significantly different (p<0.05).

Hemocyte counts in *L. vannamei* after 20 days with experimental feed showed that mean total hemocytes after treatment with Majapahit fruit powder at moderate doses of 3.04% (treatment B) and 6.08% (treatment C) increased compared to treatment D (dose 9.12%). Thus, the addition of Majapahit fruit powder produces a significant effect (p<0.05) on hemocytes that play a role in enhancing shrimp immunity. Treatments did not differ after feeding and infection with *V. alginolyticus* and *V. parahaemolyticus;* however, after infection with *V. harveyi*, THCs varied. The most effective treatment used a dose of 3.04% (Figure-4).

### Phagocytosis activity

Phagocytosis in shrimp after 20 days with experimental feed showed that mean THCs in treatment B (dose 3.04%) increased phagocytosis by 82.27%. The increased resistance of shrimp could be due to enhanced phagocytosis (AF) by hemocytes. Phagocytosis in treatment A (dose 0%) or without the use of Majapahit fruit extract was 73.91% which lower than in treatment B (dose 3.04%). The addition of Majapahit fruit extract induces a significant increase (p<0.05) in phagocytosis. This effect enhances immune activity of shrimp and is due to antibacterial and flavonoid content in the fruit extract *(C. cujet*e L.).

After infection for 24 h, phagocytosis activity decreased 75.13%, reflected in mean values of total hemocytes in treatment B (dose 3.04%), C (dose 6.08%), and D (dose 9.12%), with the use of Majapahit fruit powder in feed. Phagocytosis in treatment A (dose 0%) or without the use of Majapahit fruit powder increased phagocytosis activity by 82.08%. The addition of Majapahit fruit powder has a significant impact on (p<0.05) phagocytosis activity.

**Figure-5:**
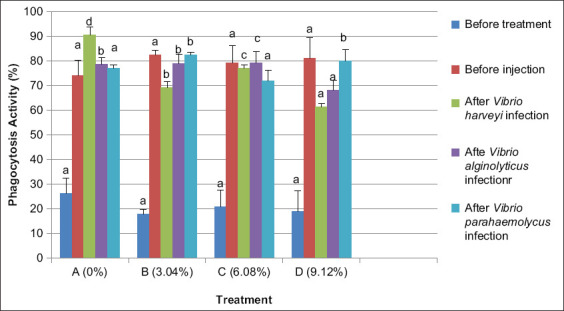
Phagocytosis activity of vannamei shrimp (*Litopenaeus vannamei*) before and after infected with *Vibrio harveyi, Vibrio alginolyticus*, and *Vibrio parahaemolyticus* and treatment with Majapahit (*Crescentia cujete* L.) fruit powder. Data were indicated as mean value±SD. Graphs with different notations showed significantly different (p<0.05).

No significant difference after feeding with Majapahit fruit powder in feed was observed. However, the highest phagocytosis activity after being infected with *V. harveyi* and *V. alginolyticus* was observed for treatment C (dose 6.08%). After infection with *V. parahaemolyticus*, the highest activity was found for treatment B (dose 3.04%) (Figure-5).

## Discussion

Majapahit fruit powder is absorbed by shrimp and induces hemocyte cells to degranulate and increase phagocytic activity. Increasing numbers of hyaline cells are related to this activity based on previous *in vitro* and *in silico* studies [10]. The use of the Majapahit plant, including the stem, leaves, and fruit, enhances the immune system in cage catfish [8] and tilapia [11].

Treatment with petal and mangrove fruit extracts on hyaline cells decreased due to infection with *Vibrio* spp. [12]. Granulocytes play a role in cellular defenses and lymphocytes produce more granulocytes than hyaline cells. The latter are activated by opsonic factors resulting from the activation of prophenoloxidase (ProPO) to phenoloxidase (PO) in granular cells. This activation enhances phagocytosis of foreign bodies, such as bacteria, viruses, and fungi.

The reduction of semi-granular cells in the present study was influenced by phagocytic activity at the area of infection. Semi-granular cells are matured hyaline cells. Hyaline cells are responsible for phagocytosis at sites of infections and do not develop into semi-granular cells. Semi-granular cells are instead involved in the encapsulation process. Encapsulation is a defense reaction against large numbers of particles that hemocyte cells are unable to phagocytize. Semi-granular cells are characterized by the presence of granules in the cytoplasm. These cells respond to polysaccharides in bacterial cell walls [13].

Granular cells undergo degranulation in the presence of pathogens. These granular cells produce PO enzymes involved in non-specific defense systems. These enzymes are responsible for production and secretion of toxic metabolites [9]. Increasing numbers of hemocytes are assumed to be part of cellular immune responses in shrimp since hemocytes are known to be involved in defense mechanisms [14]. *Sagarsum duplicatum* extract was able to increase the number of hemocytes in vannamei shrimp (*L. vannamei*) either by immersion or injection [15]. Hemocytes are synthesized by hematopoietic tissue in a pair of epigastric nodules located on the back of the stomach and involved in hemocyanin synthesis. Production of hemocytes reaches homeostasis after the introduction of immunostimulants. If immunostimulants increase hemocyanin, hemocyte counts increase directly [9].

Total hemocytes in *L. vannamei* fed with the addition of Majapahit fruit powder were reduced the total value of hemocytes after infection with *V. harveyi, V. alginolyticus*, and *V. parahaemolyticus* under treatment C (dose 6.08%). Hemocyte cells in the presence of pathogens degranulate and induce cytotoxicity and lysis of invading cells. Numbers of hemocytes in hemolymph will decrease because blood cells will migrate to infected areas. Total hemocytes decreased after *Vibrio anguillarum* infection due to THC reduction. This effect reflects defensive activity. Hemocytes are crucial for immune function and destroy particles and foreign objects that enter the shrimp hemocoel through phagocytosis, encapsulation, nodular aggregation, melanization, cytotoxicity, and intercellular communication [13]. Hemocytes are also involved in wound management through initiation of coagulation processes by producing and releasing the ProPO system [16]. According to Rodrigues and Le Moullac [17], hemocytes play a role in the formation and degradation of important molecules in hemolymph, such as a_2_-macroglobulin, agglutinins, and antimicrobial peptides.

Treatment C (dose 6.08%) with Majapahit fruit powder reduced THCs by 6.3×10^6^ cells/mL after infection with *V. alginolyticus*. Song *et al*.[18] indicated the hypoxic conditions reduce and sensitize hemocytes after infection with *V. alginolyticus*. Treatment D (dose 9.12%) after such infection produced an increase in hyaline cell counts of 15.9×10^6^ cells/mL. Thus, infection by pathogenic bacteria increases THC. A similar result was previously reported by Anderson and Siwicki [19]. Inclusion of Majapahit fruit extract at levels that exceed the body’s ability to respond will cause immunosuppression and disrupt shrimp immunity [19]. Addition of Majapahit fruit extract at different doses induces significant effects (p<0.05) on total hemocytes and may help terminate *Vibrio* spp. infection.

The antibacterial content in earthworms increased hemolymph phagocytosis activity in *L. vannamei* [20]. According to Chifdhiyah [21], the addition of white turmeric extract (*Kaempferia rotunda*) increased phagocytosis activity in tiger prawns. Majapahit fruit extract contains a bioactive flavonoid associated with phagocytic activity. Further, the antioxidant content of flavonoids can increase phagocytic cell activity [22]. The mechanism of antimicrobial action of flavonoids may include inhibition of (1) nucleic acid synthesis, (2) cell membrane function, and (3) energy metabolism [23].

Phagocytosis begins with the attachment and ingestion of microbial particles onto phagocytic cells. Phagocytic cells then form digestive vacuoles called phagosomes. A lysosome then fuses with the phagosome to form phagolysosome. Microorganisms are destroyed from the inside through egestion [21]. A reduction in phagocytic activity after infection with bacteria *V. harveyi*, *V. alginolyticus*, and *V. parahaemolyticus* indicates that phagocytic cells in hemolymph migrated to the infected area in response to the infection. Thus, phagocytic activity decreased. Phagocytic cells are reduced after infection with *V. anguillarum* due to cellular immune responses [14]. Finally, giving fruit peel extract can inhibit the growth of *V. alginolyticus* [23].

## Conclusion

The addition of Majapahit fruit powder to feed formulas might enhance cellular immune response in *L. vannamei*. The best doses of Majapahit fruit powder ranged from 3.04% to 6.08%.

## Authors’ Contributions

SR: Designed the research and wrote the manuscript. RA: Conducted the study and analyzed the data. HP: Interpreted the results and reviewed the manuscript. All authors read and approved the final manuscript.
